# The Possible Role of Electrical Stimulation in Osteoporosis: A Narrative Review

**DOI:** 10.3390/medicina59010121

**Published:** 2023-01-08

**Authors:** Weifei Zhang, Yuanrui Luo, Jixuan Xu, Chuan Guo, Jing Shi, Lu Li, Xiao Sun, Qingquan Kong

**Affiliations:** 1 Department of Orthopedics, Orthopedic Research Institute, West China Hospital, Sichuan University, Chengdu 610041, China; 2 Hospital of Chengdu Office of People’s Government of Tibetan Autonomous Region (Hospital C T), Sichuan University, Chengdu 610041, China; 3 Department of Orthopedics, Hospital of Chengdu Office of People’s Government of Tibetan Autonomous Region (Hospital C T), Orthopedic Research Institute, West China Hospital, Sichuan University, Chengdu 610041, China

**Keywords:** electrical stimulation, osteoporosis

## Abstract

Osteoporosis is mainly a geriatric disease with a high incidence, and the resulting spinal fractures and hip fractures cause great harm to patients. Anti-osteoporosis drugs are the main treatment for osteoporosis currently, but these drugs have potential clinical limitations and side effects, so the development of new therapies is of great significance to patients with osteoporosis. Electrical stimulation therapy mainly includes pulsed electromagnetic fields (PEMF), direct current (DC), and capacitive coupling (CC). Meanwhile, electrical stimulation therapy is clinically convenient without side effects. In recent years, many researchers have explored the use of electrical stimulation therapy for osteoporosis. Based on this, the role of electrical stimulation therapy in osteoporosis was summarized. In the future, electrical stimulation might become a new treatment for osteoporosis.

## 1. Introduction

Osteoporosis, a systemic bone disease, can cause fractures due to a decrease in bone density and bone quality for various reasons, the destruction of bone microstructure, and an increase in bone fragility [1,2,3]. The diagnosis of osteoporosis mainly relies on dual energy X-ray absorptiometry, and the main measurement sites are the lumbar spine, proximal femur, and distal radius. Generally speaking, the measurement value is less than −2.5 [4,5], and the incidence of osteoporosis is very high. In a community survey of 5585 people with an average age of 77 years old in the UK, it was found that the incidence of osteoporosis was more than 50% [6]. In osteoporosis patients, spine fractures and hip fractures are common; they affect the quality of life of patients, threaten the health of elderly patients, and can cause fatal complications [7,8,9]. Osteoporosis treatments include bisphosphonates, selective estrogen receptor modulators, estrogens, and parathyroid hormones. However, some of these drugs are clinically inconvenient to use, and most of them have side effects, so it is clinically meaningful to develop new osteoporosis treatments [10,11,12,13,14].

**Figure 1:**
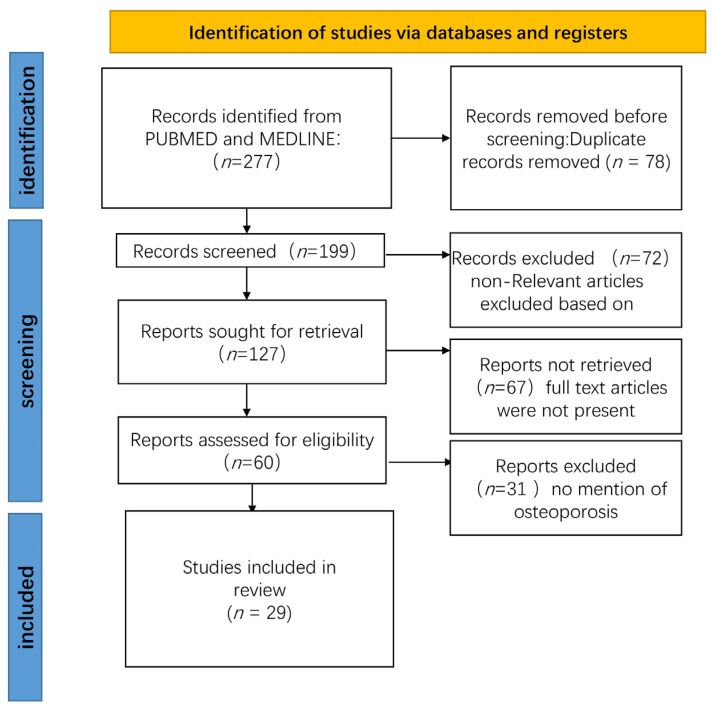
PRISMA flowchart showing the process of study selection.

Electrical stimulation therapy mainly includes pulsed electromagnetic fields (PEMFs), direct current (DC), and capacitive coupling (CC) [15,16,17] (Figure 1). The main working principle of a PEMF is to convert electric current into electromagnetic wave signal, and a PEMF has curative effect on nonunion and delayed fracture healing [18,19,20]. DC is an invasive technique that delivers constant direct current through an implanted electrical stimulator [21,22,23]. CC is a non-invasive technology that applies an electric field, and two skin electrodes are aimed at the treatment site. Therefore, electrical energy is induced from one electrode to the other electrode through frequency changes to stimulate the treatment site and achieve the purpose of treatment [24,25,26]. Studies have reported that a PEMF, DC, and CC have therapeutic effects on osteoporosis. Based on this, the relevant literature was reviewed to provide information for new treatment options for osteoporosis.

## 2. Methods

We used “electrical stimulation”, “osteoporosis”, “pulsed electromagnetic fields”, and “osteoporosis” as keywords and searched all the articles from 1968 to 2022 in PUBMED and MEDLINE articles. We included only publications published in English and selected those findings that were, in our opinion, the most important. We further analyzed these articles, mainly selected papers from the past 5 years, but also included well-respected older publications (Figure 1).

## 3. PEMFs for Osteoporosis

The main mechanism of a PEMF is to convert electric current into a magnetic field that can activate the biological current of the organism to achieve the purpose of treatment. A PEMF has good effects on nonunion and delayed fracture healing, and there are also many reports that a PEMF can treat osteoporosis [27,28,29]. The clinical research into PEMFs in osteoporosis, the effects of a PEMF on osteogenesis and its mechanism, and the effects of a PEMF on osteoclasts and its mechanism will be discussed.

### 3.1. The Clinical Research into PEMFs in Osteoporosis

The improvement of osteoporotic bone pain and osteoporosis using PEMFs have been reported on many times (Table 1).

At present, there is clinical evidence that PEMFs could relieve osteoporotic bone pain [30]. Many clinical trials have proved that PEMFs can promote the recovery of osteoporotic bone mass. Antonino Catalano et al. conducted a randomized controlled clinical trial of 43 people and revealed that a PEMF may play a role in restoring osteoporosis and bone mass through RANKL/OPG and Wnt/β-catenin pathways [31]. Of course, other clinical studies have come to similar conclusions [32,33]. Although the FDA (Food and Drug Administration) has not yet approved PEMFs for the treatment of osteoporosis, the above clinical trials have indicated that PEMFs are a non-invasive, safe, and effective treatment for osteoporosis. We look forward to more large-scale clinical trials using PEMFs for osteoporosis in the future. Through further trials, PEMFs would be approved by the FDA for osteoporosis treatment in the future, which will benefit patients; however, this technology is not mature enough for clinical use and needs more research

**Table 1:** The clinical research of PEMFs in osteoporosis.

Researchers	Contents	Patients	References
Antonino Catalano et al.	restoring osteoporosis and bone mass	43	[31]
Hui-Fang Liu et al.	as effective as alendronate	44	[32]
F Tabrah et al.	prevention and treatment of osteoporosis	20	[33]

### 3.2. The Effects of a PEMF on Osteogenesis and Its Mechanism

A PEMF inhibits osteoporosis progression mainly through primary cilia, H-type angiogenesis, and WNT signaling pathways (Table 2).

Cilia are hair-like organelles protruding from the cell surface and are composed of microtubules to form axons. Furthermore, cilia play an important role in the basic life activities of cells. Immobile cilia are also commonly referred to as primary cilia; they sense changes in the microenvironment surrounding the cell and mediate a variety of important signal transductions within the cell [34,35,36]. Wen-Fang He et al. revealed that PEMFs promote osteogenic differentiation through nitric oxide signaling within primary cilia, thus inhibiting osteoporosis progression [37]. According to Yan-Fang Xie et al., PEMFs stimulated osteoblast differentiation and mineralization in a primary cilia-dependent manner and inhibited osteoporosis progression [38]. Juan-Li Yan et al. illustrated that PEMFs promote osteoblast mineralization and maturation through primary cilia as a potential treatment for osteoporosis [39]. Therefore, it can be said that primary cilia play an important role in the mechanism of PEMF treatment of osteoporosis.

There are special capillaries in the bone—H-type blood vessels—that are CD31 protein-positive vessels, and H-type angiogenesis is also considered as a key factor in osteoporosis [40,41,42,43]. Qian Wang et al. found that PEMFs promote osteogenesis by promoting the formation of H-type blood vessels which inhibits the occurrence of osteoporosis [44]. Tiantian Wang et al. also found that, as a potential treatment for glucocorticoid-induced osteoporosis, PEMFs maintain H-type angiogenesis and osteogenesis [45].

The WNT pathway is an evolutionarily conserved signaling pathway that is involved in various biological activities. It can be divided into a canonical Wnt/β-catenin signaling pathway, planar cell polarity pathway, Wnt/Ca+ pathway, and spindle regulation pathway [46,47,48,49,50]. The WNT signaling pathway plays an important role in promoting osteogenesis and inhibiting osteoporosis as well [51,52,53]. Based on evidence from Xi Shao et al., PEMFs treat osteoporosis in type 2 diabetic mice by activating Wnt/β-catenin signaling [54]. Shaoyu Wu et al. found that PEMFs promote the osteogenic differentiation of mesenchymal stem cells through the Wnt/Ca+ pathway and inhibit the progression of osteoporosis [55]. Xi Shao et al. discovered that PEMFs enhance canonical Wnt signaling-mediated bone formation in spinal cord injured rats [56]. In addition, other researchers have also revealed that PEMFs inhibit the progression of osteoporosis through the WNT signaling pathway [57,58].

**Table 2:** The effects of a PEMF on osteogenesis and its mechanism.

Researchers	Outcome	Mechanism	References
Wen-Fang He et al.	osteogenic differentiation	primary cilia	[37]
Yan-Fang Xie et al.	osteoblast mineralization	primary cilia-dependent	[38]
Juan-Li Yan et al.	osteoblast maturation	primary cilia	[39]
Qian Wang et al.	osteogenesis	H-type blood vessels	[44]
Tiantian Wang et al.	osteogenesis	H-type angiogenesis	[45]
Xi Shao et al.	osteogenesis	Wnt/β-catenin	[54]
Shaoyu Wu et al.	osteogenic differentiation	Wnt/Ca+	[55]
Xi Shao et al.	bone formation	Wnt/β-catenin	[56]
Da Jing et al.	bone formation	Wnt/β-catenin	[57]
Jun Zhou et al.	osteogenesis	Wnt/β-catenin	[58]

### 3.3. The Effects of a PEMF on Osteoclasts and Its Mechanism

PEMFs can also inhibit the progression of osteoporosis by affecting osteoclasts (Table 3). Pan Wang et al. found that PEMFs inhibit osteoclast formation by regulating the ratio of RANKL/OPG through primary cilia, thus inhibiting the progression of osteoporosis [59]. Ying Pi et al. found that a low-frequency PEMF inhibits osteoclast differentiation by scavenging reactive oxygen species, and it is a potential treatment for osteoporosis [60]. Yutian Lei et al. found that PEMFs inhibit osteoclast differentiation and inhibit the progression of osteoporosis by regulating the Akt/mTOR signaling pathway [61]. Moreover, many other researchers have found that PEMFs inhibit the progression of osteoporosis by inhibiting osteoclast formation and differentiation [62,63,64,65,66].

## 4. DC for Osteoporosis

DC is an invasive technique that provides a constant direct current by implanting an electrical stimulator and implanting the cathode and anode into the repair site for continuous electrical stimulation to treat diseases. In addition, DC also has a therapeutic effect on fracture nonunion [67,68]. At present, many animal experiments have confirmed that DC has a therapeutic effect on osteoporosis (Table 4). Kaori Iimura et al. discovered that chronic stimulation of the superior roaring nerve in rats with implanted electrodes stimulated the thyroid to release calcitonin and partially ameliorated bone loss in OVX (ovariectomy) rats [69]. Roy Yuen-Chi Lau et al. illustrated that electrical stimulation of the dorsal root ganglion of rats using an implantable micro-electrical stimulation system can stimulate the secretion of calcitonin gene-related peptides and inhibit osteoporosis as well [70]. Y-C Lau et al. also demonstrated that DC can be used to stimulate dorsal root nerve energy-saving treatment for osteoporosis in rats [71]. However, at present, DC has a more important problem, which is the potential infectivity of implanted electrical stimulation devices for patients, and researchers should take this factor into consideration in future studies.

## 5. CC for Osteoporosis

CC is a non-invasive technology that applies an electric field; two skin electrodes are aimed at the treatment site so that the electric energy is induced from one electrode to the other electrode through frequency changes, thereby stimulating the treatment site and achieving the purpose of treatment. Furthermore, the current therapeutic role of CC in fractures and bone pain has also been reported [72,73]. The therapeutic effects of CC in osteoporosis are also limited to animal experiments (Table 5). Jayanand Manjhi et al. found that low-level capacitively coupled pulsed electric field stimulation has a therapeutic effect on osteoporosis in rats [74]. Jayanand Behari et al. revealed that capacitively coupled stimulation of rat legs can inhibit the progression of osteoporosis [75]. C T Brighton et al. proved that the stimulation of vertebrae at the paraspinal level using capacitively coupled electrodes on the dorsal skin surface of the 11th thoracic and 4th lumbar vertebrae treats osteoporosis [76]. Other researchers have also obtained similar conclusions [77,78]. In general, CC is a promising treatment modality, with good efficacy in animal models, and more clinical trials are needed to confirm this.

## 6. Conclusions

Electrical stimulation might be a good, non-invasive, and effective treatment for osteoporosis. A PEMF is the treatment method with the most research and many clinical trials. Many clinical trials have also shown its efficacy, but the specific treatment parameters of frequency and treatment time are not yet conclusive. Meanwhile, the FDA has not approved the treatment for osteoporosis, and even though PEMFs have huge potential, PEMFs need more large-scale clinical trials in the future to investigate the optimal frequency and duration of treatment for osteoporosis. DC is an invasive therapy. At present, only animal experiments have proved its efficacy, and researchers need to solve the potential infection problems of DC before going to clinical trials. CC is also a promising treatment for osteoporosis, and multiple animal experiments have confirmed its efficacy; however, it still needs clinical trials to confirm its effects on osteoporosis patients.

Our study has limitations. The first is the small number of cases included in the study, and the conclusions drawn may have limitations. The second is that the exact magnitude and frequency of the effects of PEMFs, CC, and DC were not determined, which might pose difficulties for future applications in the clinic.

## Figures and Tables

**Table 3:** PEMFs inhibit the progression of osteoporosis by affecting osteoclasts.

Researchers	Outcome	Mechanism	References
Pan Wang et al.	osteoclast formation	RANKL/OPG	[59]
Ying Pi et al.	osteoclast differentiation	reactive oxygen	[60]
Yutian Lei et al.	osteoclast differentiation	Akt/mTOR	[61]
Zhiming He et al.	osteoclast formation	TGF-β	[62]
Pan Wang et al.	osteoclast formation	RANKL	[63]
Jie Zhang et al.	osteoclast differentiation	Ca^2+^	[64]
Jianquan He et al.	osteoclast formation	NFATc1	[65]
Kyle Chang et al.	osteoclast formation	apoptotic rate	[66]

**Table 4:** Animal experiments using DC for osteoporosis.

Researchers	Animal	Position	References
Kaori Iimura et al.	rats	superior roaring nerve	[69]
Roy Yuen-Chi Lau et al.	rats	dorsal root ganglion	[70]
Y-C Lau et al.	rats	dorsal root ganglion	[71]

**Table 5:** Animal experiments using CC for osteoporosis.

Researchers	Animal	Position	References
Jayanand Manjhi et al.	rats	leg	[74]
Jayanand Behari et al.	rats	leg	[75]
C T Brighton et al.	rats	vertebrae	[76]
C T Brighton et al.	rats	tibia	[77]
C T Brighton et al.	rats	sciatic nerve	[78]

## Data Availability

Not applicable.
